# Removal of Chromium (VI) by *Escherichia coli* Cells Expressing Cytoplasmic or Surface-Displayed ChrB: a Comparative Study

**DOI:** 10.4014/jmb.1912.12030

**Published:** 2020-03-20

**Authors:** Xiaofeng Zhou, Jianghui Li, Weilong Wang, Fan Yang, Bingqian Fan, Chenlu Zhang, Xiaojun Ren, Feng Liang, Rong Cheng, Fengying Jiang, Huaibin Zhou, Juanjuan Yang, Guoqiang Tan, Jianxin Lyu, Wu Wang

**Affiliations:** 1 Zhejiang Provincial Key Laboratory for Technology and Application of Model Organisms, School of Laboratory Medicine and Life Sciences, Wenzhou Medical University, Wenzhou 325035, Zhejiang, P.R. China; 2 School of Public Health and Management, Wenzhou Medical University, Wenzhou 325035, Zhejiang, P.R. China

**Keywords:** Chromium (VI), ChrB, biosorption, cytoplasmic expression, surface display

## Abstract

Various genetically engineered microorganisms have been developed for the removal of heavy metal contaminants. Metal biosorption by whole-cell biosorbents can be enhanced by overproduction of metal-binding proteins/peptides in the cytoplasm or on the cell surface. However, few studies have compared the biosorption capacity of whole cells expressing intracellular or surface-displayed metal-adsorbing proteins. In this study, several constructs were prepared for expressing intracellular and surface-displayed *Ochrobactrum tritici* 5bvl1 ChrB in *Escherichia coli* BL21(DE3) cells. *E. coli* cells expressing surface-displayed ChrB removed more Cr(VI) from aqueous solutions than cells with cytoplasmic ChrB under the same conditions. However, intracellular ChrB was less susceptible to variation in extracellular conditions (pH and ionic strength), and more effectively removed Cr(VI) from industrial wastewater than the surface-displayed ChrB at low pH (<3). An adsorption- desorption experiment demonstrated that compared with intracellular accumulation, cell-surface adsorption is reversible, which allows easy desorption of the adsorbed metal ions and regeneration of the bioadsorbent. In addition, an intrinsic ChrB protein fluorescence assay suggested that pH and salinity may influence the Cr(VI) adsorption capacity of ChrB-expressing *E. coli* cells by modulating the ChrB protein conformation. Although the characteristics of ChrB may not be universal for all metal-binding proteins, our study provides new insights into different engineering strategies for whole-cell biosorbents for removing heavy metals from industrial effluents.

## Introduction

Heavy metals are major contributors to environmental pollution and pose serious health risks to humans. Various physicochemical treatment techniques for the removal of metal contaminants have been developed; however, they are usually energy-intensive and may produce hazardous byproducts. Recently, biological treatment techniques for the removal of toxic heavy metals have attracted much attention. Some natural biomasses (such as bacteria, fungi, and algae) have been found to be highly effective in the bioaccumulation or biosorption of heavy metals. Furthermore, microorganisms can be genetically engineered to enhance their biosorption capacity through overexpression of metal-binding proteins in the cytoplasm [1, 2] or on the cell surface [3, 4]. However, these two strategies (intra- or extracellular biosorption) have not been comparatively characterized.

Chromium (VI) is one of the most toxic environmental pollutants due to its mutagenic and carcinogenic properties. It is widely used in the stainless steel industry, electroplating, and in leather tanneries. Interestingly, a variety of microorganisms capable of removing Cr(VI) contamination and resistant to high levels of chromate can be isolated from heavy metal-contaminated environments. The resistance mechanisms mainly comprise efflux of chromate ions from the cytoplasm and reduction of Cr(VI) to the less toxic Cr(III) form [5]. *Ochrobactrum tritici* 5bvl1, isolated from chromium-contaminated wastewater, is a highly Cr(VI)-resistant bacterial strain [6]. Its tolerance to high chromate concentrations is attributed to the presence of a specific transposon, Tn*OtChr*, which harbors a group of chromate resistance genes, including *chrB*, *chrA*, *chrC*, and *chrF* [7]. As a chromate efflux pump, ChrA has been suggested to extrude chromate from the cytoplasm out of the cell, thereby lowering the intracellular Cr(VI) concentration [7]. ChrC and ChrF are putative superoxide dismutases that protect cells from reactive oxygen species (ROS) [8]. ChrB was identified as a chromate-sensing transcriptional regulator of the *chr* operon that binds directly to the *chr* promoter region [9]. In this study, we constructed different *Escherichia coli* strains that express either cytoplasmic or surface-displayed ChrB and we compared their Cr(VI)-adsorbing capacities. The results demonstrated that *E. coli* cells expressing surface-displayed ChrB were more efficient in Cr(VI) adsorption than cells expressing intracellular ChrB. In contrast, when used for treating industrial wastewater with low pH and high salinity, *E. coli* cells expressing intracellular ChrB were more effective at adsorbing Cr(VI) than those with surface-displayed ChrB. Therefore, our findings suggest that environmental factors, such as pH and ionic strength, should be considered when selecting genetically engineered microorganisms for bioremediation of heavy metals.

## Materials and Methods

### Construction of Vectors for the Expression of Intracellular or Surface-Displayed ChrB

An optimized gene sequence (GenBank Accession No. MN340684) coding for ChrB from *O. tritici* 5bvl1 (GenBank Accession No. EF469735.1) was designed by taking the codon preference of *E. coli* into consideration, and was synthesized by GenScript Biotech Corp. (China). For the constructing the plasmid construct expressing surface-displayed ChrB (pET28-*lpp-ompA-chrB* or pET-LOChrB), three DNA fragments encoding the N- terminal (amino acids 1–29) of prolipoprotein (Lpp) (EcoGene Accession No. EG10544), residues 45–159 of the outer-membrane protein OmpA (EcoGene Accession No. EG10669), and the optimized *chrB* sequence were PCR-amplified from the genomic DNA of *E. coli* JM109 or synthesized vector using the primers Lpp-1/Lpp-2, OmpA-1/OmpA-2, and LOChrB-1/LOChrB-2, respectively. The primers Lpp-1 and LOChrB-2 were used to join the DNA sequences by recombinant PCR. Using an In-Fusion HD Cloning Kit (Clontech, USA), the *lpp-ompA- chrB* PCR product was subcloned into the expression plasmid pET28b (Invitrogen, USA), which had been predigested with the restriction enzymes NcoI and XhoI. To synthesize the plasmid construct expressing intracellular ChrB, a PCR product coding for the entire ChrB gene was amplified using the primers ChrB-1/ChrB- 2 and was cloned into NcoI/XhoI-digested pET28b to generate the plasmid pET-ChrB. A plasmid construct expressing ChrB with a C-terminal His-tag (pET-ChrB-His) was constructed in a similar manner, except that ChrB-2-His (in which the stop codon was removed) was used as the downstream primer. All primers used are listed in Table S1 in the Supplementary Material.

### Induction and Purification of ChrB Protein

*E. coli* BL21(DE3) cells containing the plasmid pET-ChrB-His (pET-ChrB-His/BL21) were grown in Luria– Bertani (LB) medium to an optical density at 600 nm (OD_600_) of 0.6–0.7. Then, 200 μM isopropyl β-D- thiogalactoside (IPTG) was added to induce protein expression at 25°C for 24 h. His-tagged ChrB (ChrB-His) was purified using a Ni-agarose column (Qiagen, Germany) as described by Branco and Morais [9], followed by desalting on a HiTrap desalting column (5 ml, GE Healthcare, USA) to remove the imidazole. The purity of the purified protein was >95% judging from the SDS-PAGE gel stained with Coomassie brilliant blue. The protein concentration of purified ChrB-His was measured at 280 nm using an extinction coefficient of 34.05 cm^–1^ mM^–1^.

### Chromium Content Measurement

Protein samples were digested and cell suspensions were lysed by microwave digestion before the analysis of chromium contents, whereas aqueous solutions were analyzed directly. The total Cr content was determined by inductively coupled plasma mass spectrometry. The concentration of Cr(VI) in these samples was measured by colorimetry using 1,5-diphenylcarbazide as an indicator [10]. The Cr(III) concentration was calculated by subtracting the Cr(VI) concentration from the total Cr concentration.

### SDS-PAGE/Western Blot Analysis of Intracellular and Surface-Displayed ChrB

*E. coli* BL21(DE3) cells expressing intracellular ChrB without His-tag (pET-ChrB/BL21) or surface-displayed ChrB (pET-LOChrB/BL21) were grown in LB medium at 37°C to an OD_600_ of 0.6–0.7, and then, protein expression was induced by the addition of 200 μM IPTG. After a 24-h incubation at 25°C, the cells were harvested and resuspended in PBS at an OD_600_ of 10. Expression of cytoplasmic and surface-displayed ChrB was detected by SDS-PAGE and western blotting using an anti-ChrB monoclonal antibody (produced by GenScript Biotech Corp.). To verify the surface display of ChrB, cells were treated with trypsin (200 μg/ml final) at 37°C for 1 h. The reaction was stopped by washing the cells twice with PBS, and ChrB was then analyzed by SDS-PAGE and western blotting. Band intensity on the SDS-PAGE gel or western blot film was analyzed using ImageJ software.

### Determination of Cr(VI) Adsorption Capacity of *E. coli* Expressing Intracellular or Surface-Displayed ChrB

*E. coli* cells (pET/BL21, pET-ChrB/BL21, and pET-LOChrB/BL21) were grown in LB medium at 37°C to an OD_600_ of 0.6–0.7, and then, protein expression was induced by the addition of 200 μM IPTG. The pET/BL21 and pET-LOChrB/BL21 cells were then incubated at 25°C for 24 h, whereas the pET-ChrB/BL21 cells were harvested after 6 h (pET-ChrB-6h/BL21) or 24 h (pET-ChrB-24h/BL21) of incubation. The cells were washed with deionized water at least three times and then resuspended in 200 ml of water containing Cr^6+^ (0, 0.005, 0.025, 0.125, 0.5, 1.0, 2.5, and 5.0 mM, respectively) at OD_600_ = 10, and incubated at 25°C for 4 h. Then, the cells were harvested by centrifugation and analyzed for Cr(VI) content as described above. Cr(VI) adsorption capacity (μmol/g) was defined as the amount of Cr(VI) bound per gram (dry weight) of bacterial cells, which can be measured after lyophilization.

To further verify Cr(VI) removal capability, IPTG-induced *E. coli* cells (pET/BL21, pET-ChrB-6h/BL21, pET-ChrB-24h/BL21, and pET-LOChrB/BL21) were resuspended in 0.5 mM Cr^6+^ solution (200 ml, final cell densities at OD_600_ = 2, 5, and 10) and incubated at 25°C. Cell suspensions were collected every 30 min for 4 h and centrifuged, and Cr(VI) concentrations in the supernatants were determined.

### Comparison of Cr(VI) Resistance of *E. coli* Expressing Intracellular or Surface-Displayed ChrB

Overnight cell cultures (pET/BL21, pET-ChrB/BL21, and pET-LOChrB/BL21) were diluted 1:100 in fresh LB medium supplemented with kanamycin (50 μg/ml) and Cr^6+^ solution (0.001, 0.01, 0.05, 0.1, 0.25, 0.5, 1.0, 2.5, 5.0, or 10 mM final). IPTG (100 μM) was added to induce ChrB expression. Cell samples were adjusted to an OD_600_ of 0.02 and then grown at 25°C with vigorous shaking (250 rpm) for 12 h. Then, the cell density was measured again.

### Effect of pH and Salinity on the Cr(VI) Adsorption Capacity of *E. coli* Expressing Intracellular or Surface- Displayed ChrB

IPTG-induced *E. coli* cells (pET-ChrB-24h/BL21 and pET-LOChrB/BL21) were treated with 5 mM Cr^6+^ in 200 ml of Tris buffers with different pH values (2, 3, 4, 5, 6, 7, 8, 9, and 10) or different concentrations of NaCl (0, 0.2, 0.4, 0.6, 0.8, 1.0, 1.2, and 1.4 M, pH 7). All cell suspensions were adjusted to OD_600_ = 10. After 4 h of incubation at 25°C, the Cr(VI) adsorption capacity of each cell sample was analyzed.

### Removal of Cr(VI) from Industrial Wastewater by *E. coli* Expressing Intracellular or Surface-Displayed ChrB

A total of 12 wastewater samples were collected from chromium electroplating factories or tanneries located in the LongWan industrial district of Wenzhou (Southeast China). The samples were centrifuged (12,000 ×*g*, 10 min) to remove any undissolved particles, and the supernatants were transferred to new tubes for the Cr(VI) removal experiment. Before treatment with *E. coli* cells, the Cr(VI)/Cr(III) concentration, pH, and total dissolved solid (TDS) level of each sample were measured. TDS was determined using a gravimetric method as described previously [11]. *E. coli* cells expressing surface-displayed ChrB (pET-LOChrB/BL21) or intracellular ChrB (pET- ChrB-24h/BL21) were suspended directly in the water samples (50 ml, OD_600_ = 50). After 4 h of incubation at 25°C, the cells were removed by centrifugation (12,000 ×*g*, 10 min). The Cr(VI)/Cr(III) concentrations in the treated wastewater samples (supernatant fractions) were analyzed as described above.

### Desorption of Cr(VI) and Regeneration of *E. coli* Expressing Intracellular or Surface-Displayed ChrB

To test the regeneration capability of *E. coli* cells expressing intracellular or surface-displayed ChrB for Cr(VI) adsorption, cells expressing intracellular or surface-displayed ChrB (induced for 24 h, OD_600_ = 10) were treated with 5 mM Cr^6+^ in Tris buffer (pH 7.0) for 4 h. The Cr(VI)-treated cells were collected by centrifugation and washed with Tris buffers (pH 3, 4, 5, 6, and 7) at least twice. The total Cr(VI) content in the cell pellets was measured, and the amount of Cr(VI) desorbed was calculated as the difference between the total Cr(VI) contents of the Cr(VI)-treated cells before and after washing. After Cr(VI) desorption at the optimal pH, cells were treated with 5 mM Cr^6+^ (in Tris buffer, pH 7.0) and were again analyzed for Cr(VI) adsorption ability, which was determined as the difference between the Cr(VI) concentrations of the Cr^6+^ solutions before and after incubation with regenerated cells. At least 4 cycles of adsorption-desorption were conducted.

### Intrinsic Fluorescence Measurement

The purified ChrB protein (10 μM in buffer containing 20 mM Tris-HCl and 0.5 M NaCl) was adjusted to different pH values (2, 3, 4, 5, 6, 7, 8, 9 and 10, respectively) using concentrated HCl or NaOH. The intrinsic fluorescence of protein samples in different buffer solutions was measured in 2 mm path-length quartz microcuvettes using the Hitachi F-7000 fluorescence spectrophotometer. After all the samples were neutralized to pH ≈ 7, the intrinsic fluorescence of each sample was analyzed again. In addition, the ChrB protein samples (10 μM in buffer containing 20 mM pH 7.0 Tris-HCl and 0, 0.5, 1.0, 1.5, 2.0, and 2.5 M NaCl, respectively) were also subjected to intrinsic fluorescence measurement. Then, all the protein samples were dialyzed into the buffer without NaCl (20 mM Tris-HCl, pH 7.0) before measuring the intrinsic fluorescence of each sample once more. The emission spectra were monitored from 300 to 500 nm with the excitation at 280 nm. The spectrum of the buffer solution was used as a blank control.

## Results

### Comparison of Cr(VI) Adsorption Capacity of *E. coli* Expressing Intracellular or Surface-Displayed ChrB

**Fig. 1:**
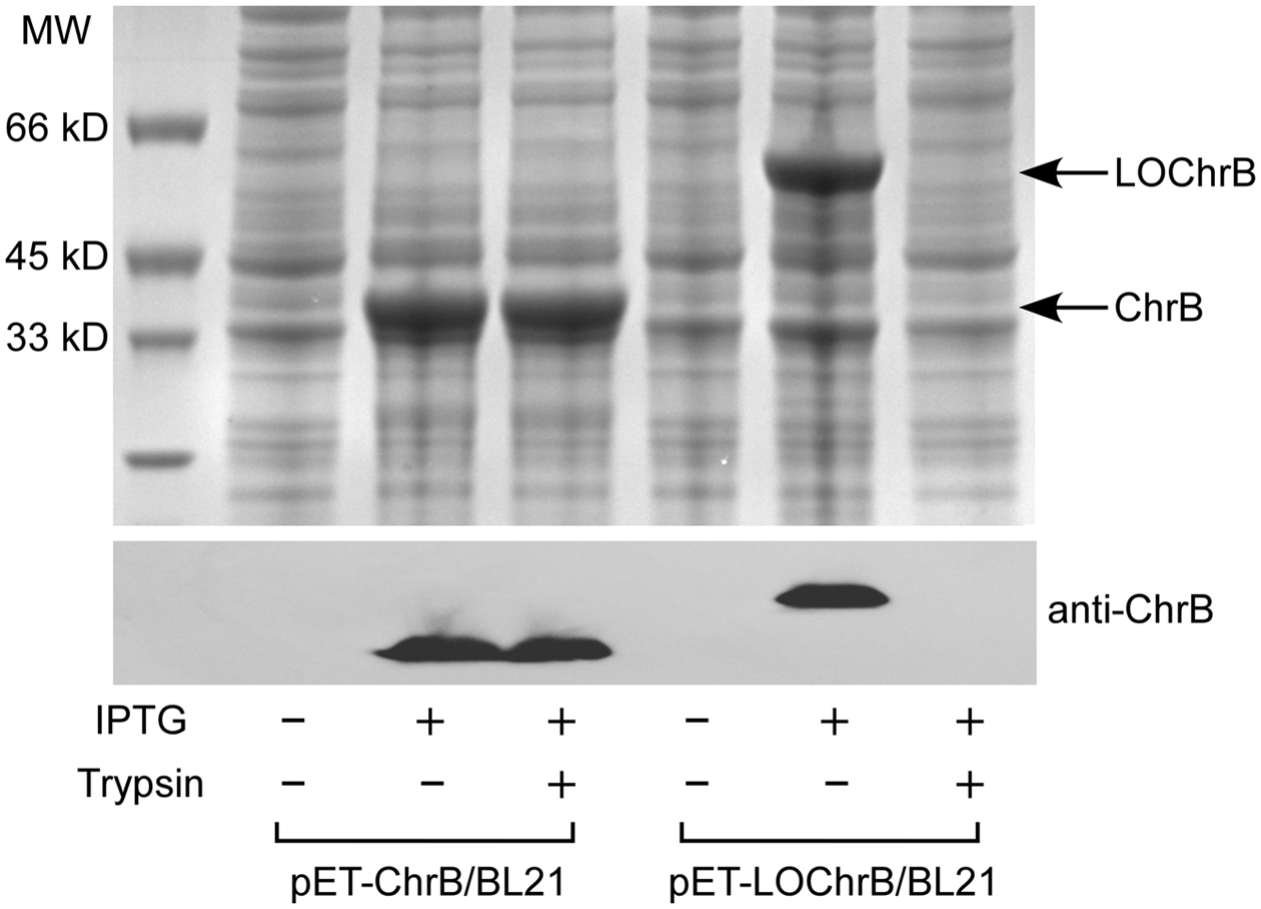
Fig. 1. SDS-PAGE gel (upper panel) and western blot (lower panel) of intracellular and surface-displayed ChrB expressed by pET-ChrB/BL21 and pET-LOChrB/BL21 cells, respectively. Cells were also treated with 100 μg/ml trypsin to verify the subcellular localization of ChrB. Results are representative of three independent experiments.

Effective removal of toxic heavy metal ions from the environment using microorganisms is usually achieved by genetic engineering approaches, which can greatly enhance the metal adsorption capacity through overproduction of metal-binding proteins in the cytoplasm or on the cell surface. In this study, we constructed two *E. coli* strains for the expression of intracellular (pET-ChrB/BL21) and surface-displayed (pET-LOChrB/BL21) ChrB. As shown in Fig. 1, intracellular ChrB (calculated MW ≈ 35 kDa) and surface-displayed ChrB (ChrB fused with the membrane protein OmpA, calculated MW ≈ 51 kDa) were both overexpressed, and the identities of the two proteins were confirmed by immunoblot analysis using anti-ChrB antibody. To verify the subcellular localization of ChrB, a protease accessibility experiment was conducted. Because the protease cannot directly permeate through the cell membrane, intracellular proteins would remain intact, whereas those exposed on the cell surface would be digested. Intracellular ChrB was resistant to 1 h of protease digestion with trypsin, whereas surface-displayed ChrB was completely degraded under the same condition (Fig. 1).

**Fig. 2:**
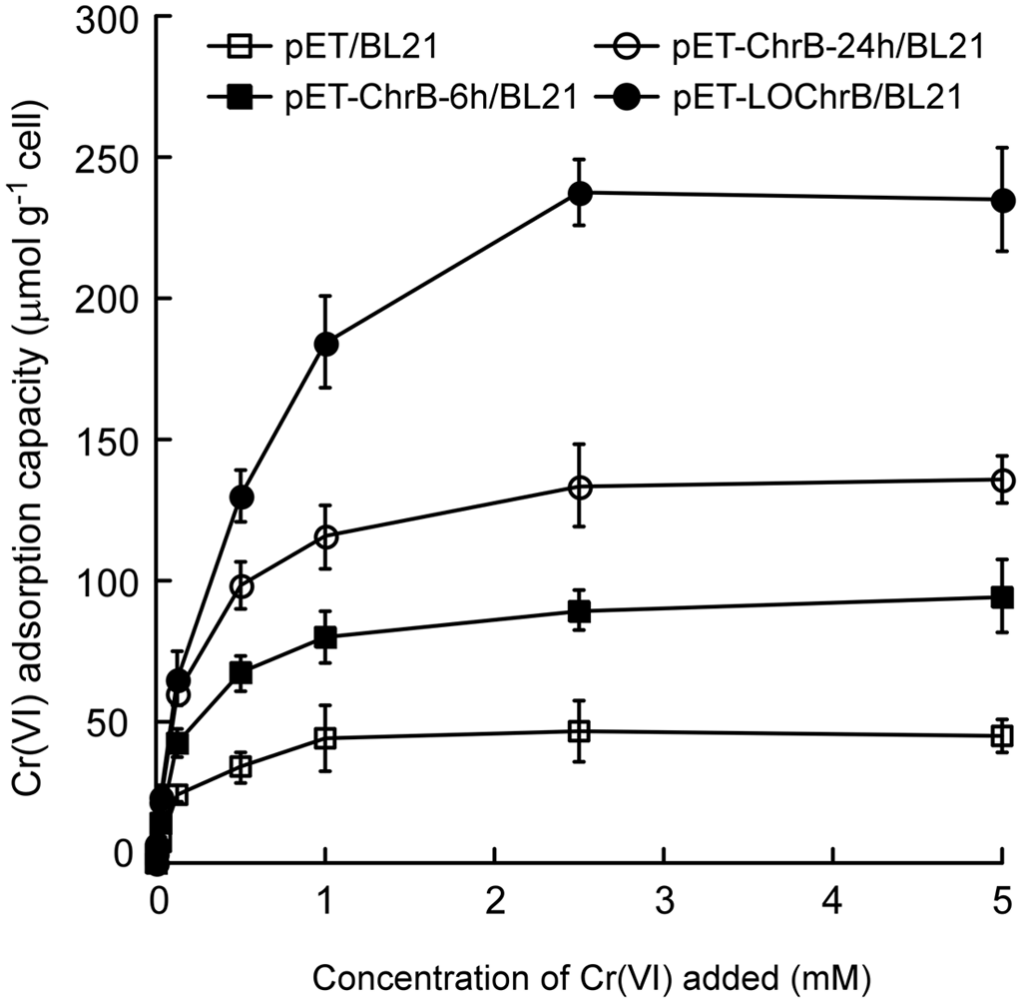
Cr(VI) adsorption capacities of *E. coli* cells expressing surface-displayed ChrB (pET-LOChrB/BL21, induced for 24 h), intracellular ChrB (6-h induction for pET-ChrB-6h/BL21 and 24-h induction for pET- ChrB-24h/BL21), and the negative control (pET/BL21). Cells were treated with increasing concentrations of Cr^6+^ (0– 5 mM). Data are the means of three independent experiments, error bars represent standard deviation.

To compare the Cr(VI) adsorption capacities of *E. coli* cells expressing intracellular or surface-displayed ChrB, an equivalent amount of protein yield per cell of both strains should be ensured. Time-course analysis of protein expression by SDS-PAGE and western blotting revealed that the amount of intracellular ChrB produced after 6 h of induction was approximately equal to that of surface-displayed protein induced after 24 h for the same cell number (calculated based on the OD_600_), and the maximal protein yields of both cell strains were achieved after 24 h of induction (Table S2). Next, the Cr(VI) adsorption capacities of four *E. coli* cell samples, pET-ChrB-6h/ BL21 (induced for 6 h), pET-ChrB-24h/BL21 (induced for 24 h), pET-LOChrB/BL21 (induced for 24 h unless indicated otherwise), and pET/BL21 (induced for 24 h unless indicated otherwise, used as the negative control), were determined. As shown in Fig. 2, the amount of Cr(VI) adsorbed increased with increasing concentration of Cr(VI) for all four cell samples and reached saturation at 2.5 mM Cr(VI). The maximum Cr(VI) content adsorbed in the three ChrB-expressing cell samples was significantly higher than that in the control. The maximum Cr(VI) adsorption capacity of pET-LOChrB/BL21 (~235 μmol/g cells) was approximately 2.5- and 1.73-fold higher than that of pET-ChrB-6h/BL21 and pET-ChrB-24h/BL21, respectively. Although pET-ChrB-24h/BL21 expressed nearly 3-fold more protein molecules per cell than pET-LOChrB/BL21, its adsorption capacity was only 57.8% of that of pET-LOChrB/BL21 under the same condition. These results suggested that ChrB expression on the cell surface greatly enhances the Cr(VI) adsorption capability of *E. coli* cells.

An artificial wastewater sample containing 0.5 mM Cr(VI), which is nearly 500 times the maximum permissible limit (0.05 mg/l or 0.96 μM) for Cr(VI) established by the United States Environmental Protection Agency (US EPA), was used to verify the Cr(VI) removal capability of *E. coli* cells expressing ChrB. As shown in Fig. S1, the Cr(VI) concentration of the water sample was decreased by 24.1%, 57.2%, and 99.1% after 2 h of treatment with pET-LOChrB/BL21 at an OD_600_ of 2, 5, and 10, respectively (Fig. S1D), whereas the same numbers of pET-ChrB-6h/BL21 cells could remove only 19.4%, 28.0%, and 42.9% of the total Cr(VI) at maximum, respectively (Fig. S1B). The above results demonstrate that *E. coli* cells expressing surface-displayed ChrB are more effective in Cr(VI) adsorption and removal from aqueous solutions than those harboring intracellular ChrB.

Additionally, the Cr(III) contents of the treated water samples were tested. As shown in Fig. S2, there were no significant differences in Cr(III) produced between the supernatant samples after treatment with pET-LOChrB/ BL21, pET-ChrB-24h/BL21, and pET/BL21 (17.9%, 16.4%, 15.6% of the original Cr(VI) content, respectively). The reduction of Cr(VI) to Cr(III) is attributed to various reductases present in the *E. coli* cells [12, 13] rather than the overexpression of ChrB. The results suggest that the removal of Cr(VI) by ChrB-engineered *E. coli* cells was mainly due to Cr(VI) adsoption rather than Cr(VI) reduction.

### Comparison of Cr(VI) Resistance of *E. coli* Expressing Intracellular or Surface-Displayed ChrB

**Fig. 3:**
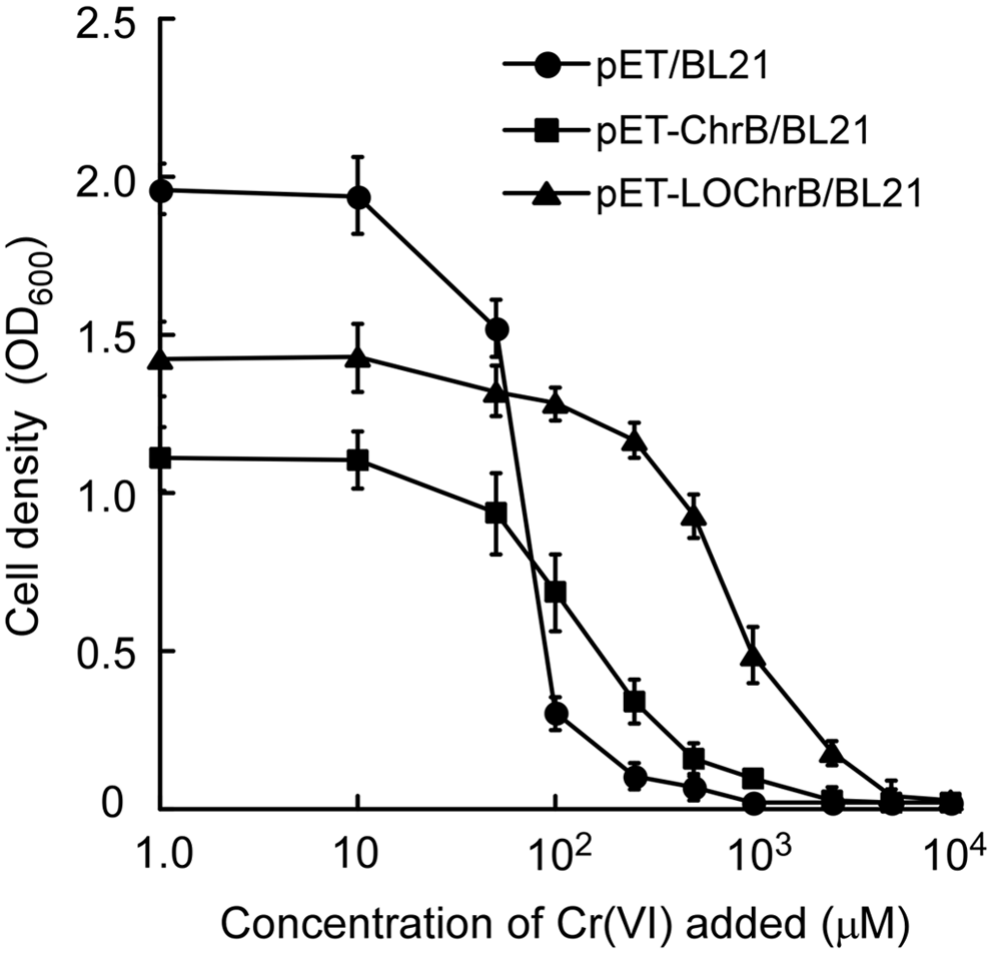
Growth of pET/BL21, pET-ChrB/BL21, and pET-LOChrB/BL21 *E. coli* cells in LB medium with increasing concentrations of Cr(VI) (0.001–10 mM) in the presence of 100 μM IPTG. The cell samples were adjusted to OD_600_ = 0.02 and then cultured at 25°C for 12 h before the OD_600_ was measured again. Data are means of three independent experiments, error bars represent standard deviation.

Generally, enhanced biosorption through overproduction of metal-binding proteins results in higher cellular tolerance to toxic metals [14]. This renders bioadsorbents more applicable for the treatment of industrial effluents, which usually contain high concentrations of heavy metals. To compare Cr(VI) resistance of *E. coli* expressing intracellular or surface-displayed ChrB, their growth in LB medium supplemented with increasing concentrations of Cr(VI) was monitored. As shown in Fig. 3, in the absence of Cr(VI), induction of intracellular as well as surface-displayed ChrB inhibited the growth of *E. coli* strains compared to that of the negative control, likely because the cells allocated energy to protein overexpression that could have been otherwise be used for cell proliferation. A low level of Cr(VI) (<10 μM) to the medium did not inhibit the growth of any of the strains. At Cr(VI) concentrations above 100 μM, cells expressing ChrB were more resistant to Cr(VI) toxicity than control cells. The minimal inhibitory concentrations (MICs) were 1 mM for pET/BL21, 2.5 mM for pET-ChrB/BL21, and 5 mM for pET-LOChrB/BL21 cells, suggesting that Cr(VI) resistance decreases in the following order: pET- LOChrB/BL21 > pET-ChrB/BL21 > pET/BL21. The increase in Cr(VI) resistance in the engineered strains can be attributed to the binding of Cr(VI) to ChrB and the concomitant alleviation of chromium stress in the cells. These results corroborate the finding that surface-displayed ChrB is more effective in Cr(VI) adsorption than intracellular ChrB.

### Effect of pH and Salinity on the Cr(VI) Adsorption Capacity of *E. coli* Expressing Intracellular or Surface- Displayed ChrB

**Fig. 4:**
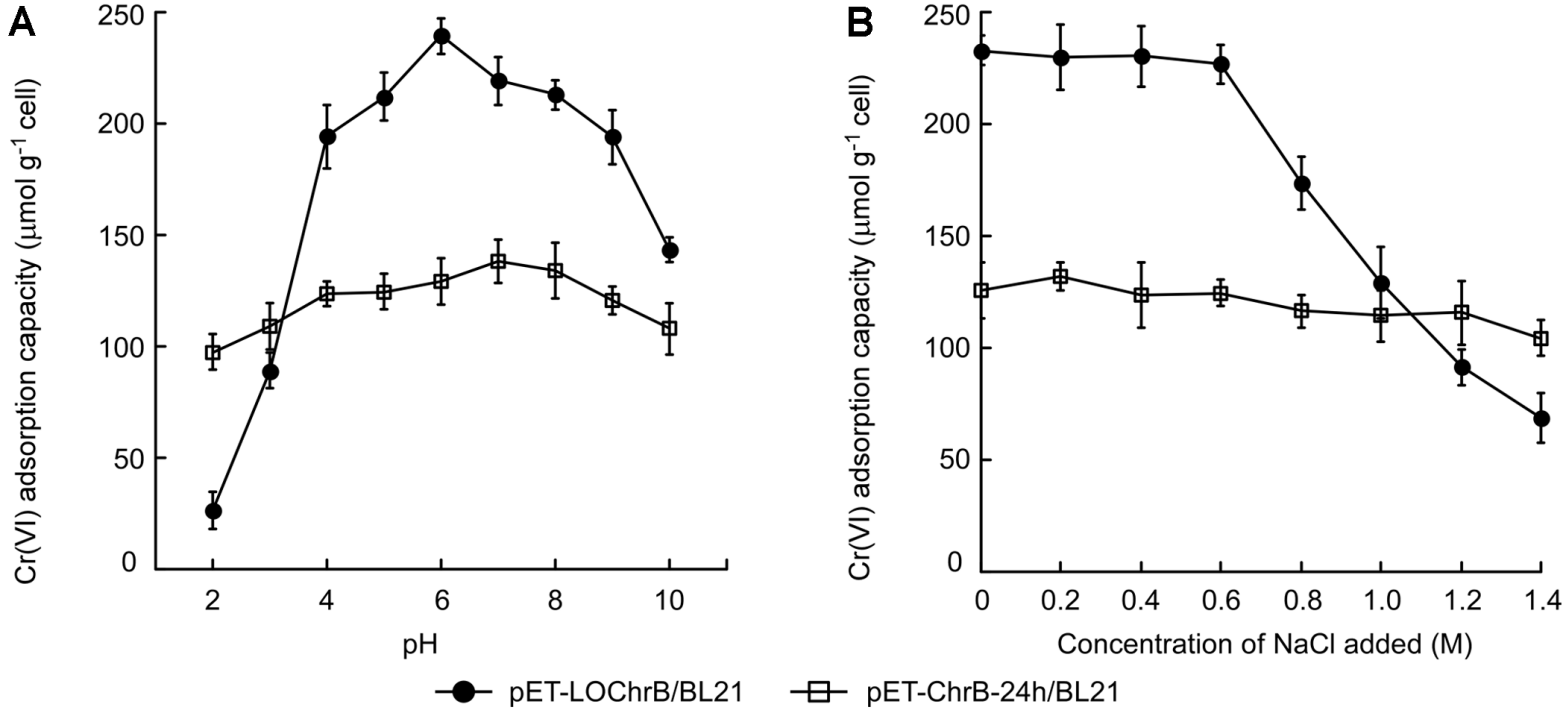
(A) Effect of pH (2–10) on the Cr(VI) adsorption capacity of *E. coli* cells expressing surface-displayed ChrB (pET-LOChrB/BL21) or intracellular ChrB (pET-ChrB-24h/BL21). (**B**) Effect of ionic strength (0–1.4 M NaCl) on the Cr(VI) adsorption capacity of *E. coli* cells expressing surface-displayed ChrB (pET-LOChrB/BL21) or intracellular ChrB (pET-ChrB-24h/BL21). Data are means of three independent experiments, error bars represent standard deviation.

Acidity (pH) considerably influences metal binding by affecting metal speciation and the structural conformation of metal-binding proteins [15, 16]. Moreover, as industrial wastewater often contains a large amount of dissolved inorganic/organic matter, the effect of other environmental factors, such as ionic strength, should also be evaluated. Thus, Cr(VI) adsorption was tested across a wide range of pH values (2–10) and NaCl concentrations (0–1.4 M). Fig. 4A depicts the pH profile of Cr(VI) adsorption by cells with intracellular (pET- ChrB-24h/BL21) and surface-displayed (pET-LOChrB/BL21) ChrB. The adsorption capacity of pET-LOChrB/ BL21 peaked at pH 6.0, and dramatically decreased under acidic conditions (pH < 4). Increased pH (pH > 8) also attenuated the Cr(VI) adsorption capacity. In contrast, Cr(VI) adsorption of pET-ChrB-24h/BL21 changed by less than 14.1% (coefficient of variation) over the pH range tested. Cr(VI) adsorption by pET-LOChrB/BL21 began to gradually decrease in the presence of NaCl at concentrations starting from 0.6 M, whereas Cr(VI) adsorption by pET-ChrB-24h/BL21 remained nearly the same at NaCl concentrations of 0–1.2 M and decreased by only 17.1% at 1.4 M (Fig. 4B). These results suggest that *E. coli* cells expressing intracellular ChrB are more resistant to low pH and high ionic strength than cells with surface-displayed ChrB. It should be noted that Cr(VI) adsorption by pET- ChrB-24h/BL21 was higher than that of pET-LOChrB/BL21 at pH ≤ 3.0 or NaCl ≥ 1.2 M.

### Removal of Cr(VI) from Industrial Wastewater Samples by *E. coli* Expressing Intracellular or Surface- Displayed ChrB

To evaluate the applicability of engineered *E. coli* cells for the removal of Cr(VI) from authentic industrial effluents, they were tested using 12 wastewater samples obtained from tanneries or chromium electroplating factories. As shown in Table S3, all samples contained high levels of Cr(VI), at varying concentrations. After treatment with pET-ChrB-24h/BL21 and pET-LOChrB/BL21 (OD_600_ = 50), the initial Cr(VI) contents of the samples decreased significantly to different extents. The slight decrease in Cr(III) was probably due to the intrinsic ability of the microorganisms to adsorb diverse metal ions in a non-specific manner [17], because ChrB was found to inefficiently bind Cr(III) (Fig. S3). Consistent with the results in Fig. 4A, pET-LOChrB/BL21 adsorbed more Cr(VI) than pET-ChrB-24h/BL21 at pH > 4, whereas it was less effective in Cr(VI) removal from highly acidic wastewater samples (pH < 3). Compared to TDS, pH strongly affected Cr(VI) adsorption by the engineered *E. coli* cells. These results suggest that for effective removal of Cr(VI) from industrial effluents, the use of either intracellular or surface-displayed ChrB-expressing strains should depend on the pH of the samples to be treated.

### Desorption of Cr(VI) and Regeneration of *E. coli* Expressing Intracellular or Surface-Displayed ChrB

**Fig. 5:**
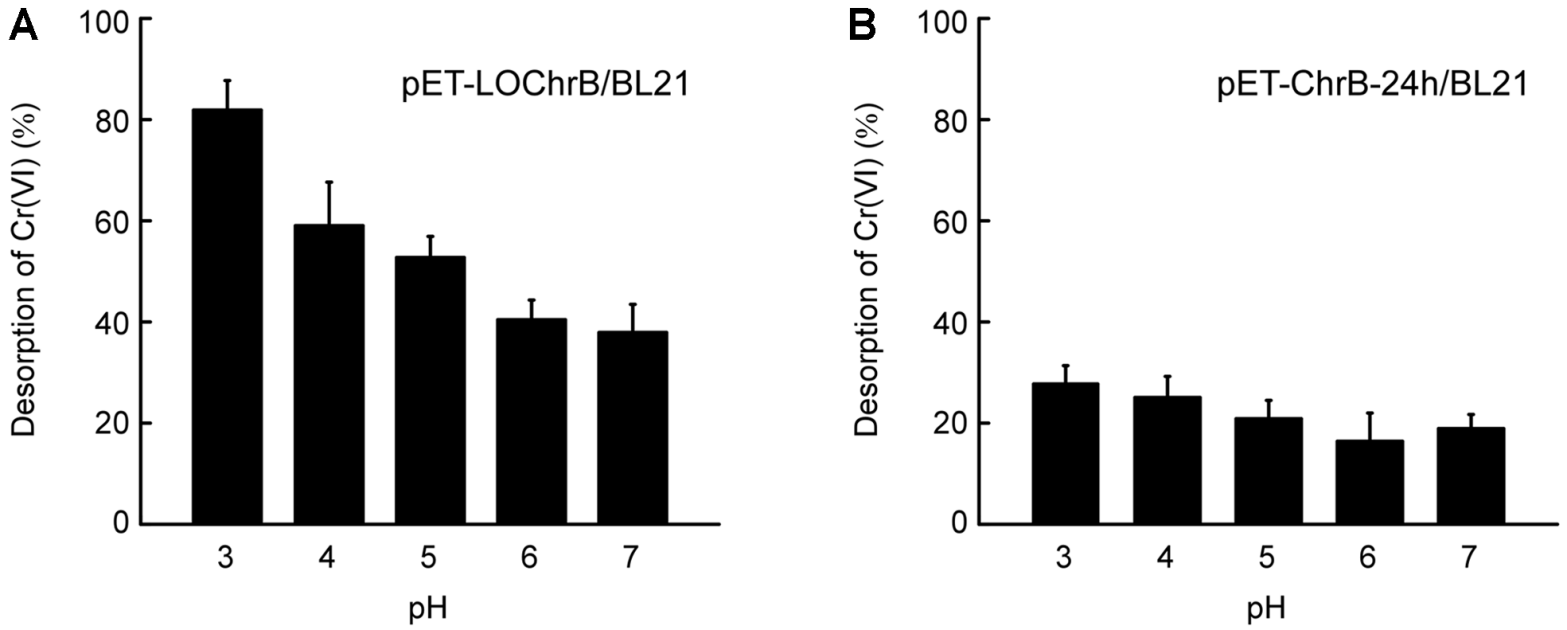
Effect of pH on Cr(VI) desorption from Cr(VI)-bound pET-LOChrB/BL21 or pET-ChrB-24h/BL21 (OD_600_ = 10) pretreated with 5 mM Cr^6+^. After at least two washes with Tris buffers of varying pH (3, 4, 5, 6, and 7), the total Cr(VI) content in each cell pellet was measured. Cr(VI) desorption was calculated as the difference between the total Cr(VI) content of the Cr(VI)-treated cells before and after washing. Data are the means of three independent experiments, error bars represent the standard deviation.

**Fig. 6:**
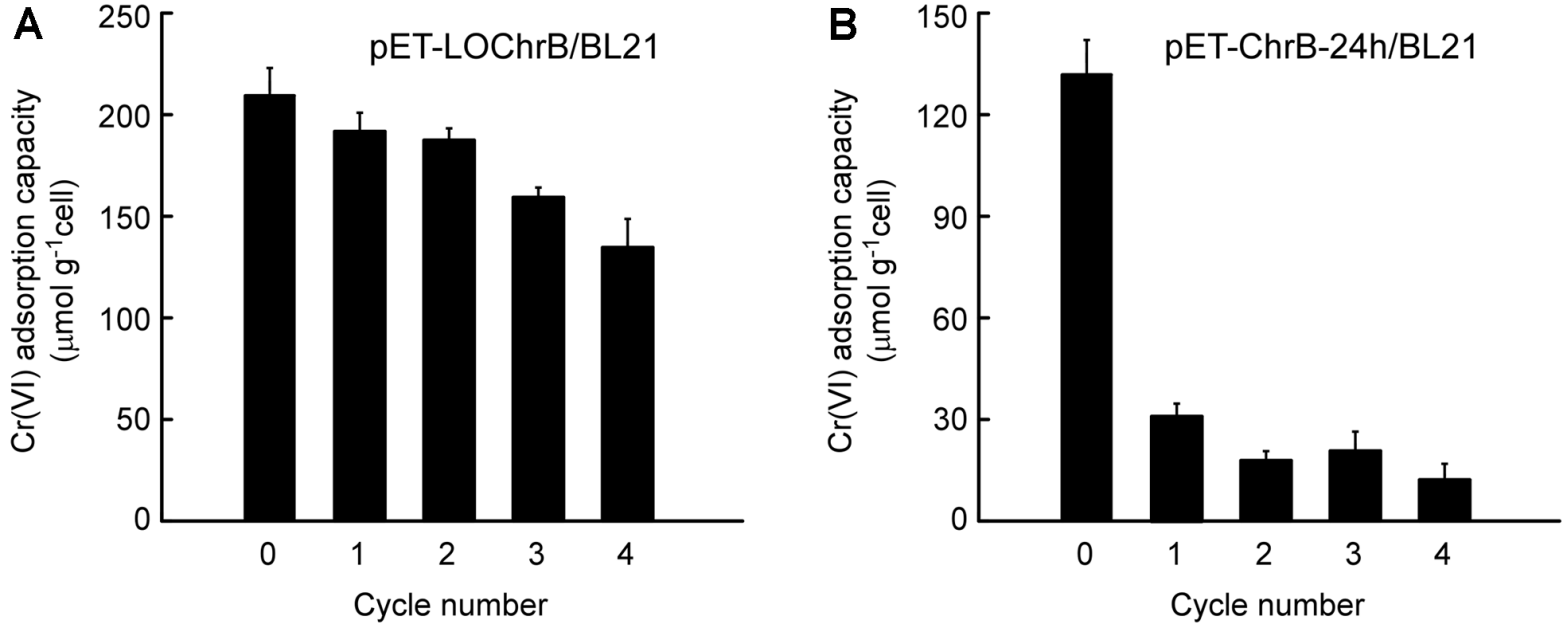
Regeneration ability of pET-LOChrB/BL21 and pET-ChrB-24h/BL21 after successive cycles of Cr(VI) adsorption and desorption. Cells (OD_600_ = 10) pretreated with 5 mM Cr^6+^ were washed with Tris buffer (pH = 3.0) at least twice to release Cr(VI) and were then treated with 5 mM Cr^6+^ to evaluate their regeneration ability. The Cr(VI) adsorption capacity of the regenerated cells was calculated as the difference between the Cr(VI) concentrations of the Cr^6+^ solutions before and after treatment with regenerated cells at the end of each of the 4 cycles. Cycle 0 represents freshly prepared cells. Data are the means of three independent experiments, error bars represent the standard deviation.

Regeneration/reuse of the bioadsorbent is usually very important with respect to both resource recovery and continuous biomass supply. Therefore, it is necessary to evaluate the regeneration ability of *E. coli* expressing intracellular or surface-displayed ChrB. The adsorption process should be followed by contaminant desorption, therefore desorption experiments using Cr(VI)-bound *E. coli* cells with intracellular or surface-displayed ChrB were performed in Tris buffers of varying pH. As shown in Fig. 5, approximately 37.4% and 18.5% Cr(VI) was released from pET-LOChrB/BL21 and pET-ChrB-24h/BL21 at pH 7.0, respectively. However, when the pH was decreased to 3.0, nearly 81.3% Cr(VI) was desorbed from pET-LOChrB/BL21 (Fig. 5A), whereas only 27.1% Cr(VI) was desorbed from pET-ChrB-24h/BL21 under the same condition (Fig. 5B). As biomass would get denatured when treated with strong acid (pH < 2.0), pH 3.0 was used to desorb Cr(VI) and regenerate the *E. coli* cells. Compared to freshly cultured pET-LOChrB/BL21, only a slight loss (<25%) in adsorption capacity was observed in regenerated cells during the first three adsorption-desorption cycles, and 64.7% binding capacity was retained at the end of the fourth cycle (Fig. 6A). In contrast, pET-ChrB-24h/BL21 could not be effectively regenerated; the Cr(VI) adsorption capacity decreased to only 23.9% when reused (Fig. 6B). These results demonstrate that compared with intracellular accumulation, cell-surface adsorption is reversible, which allows easy desorption of the adsorbed metal ions and regeneration of the bioadsorbent.

### Effect of pH and Salinity on the Protein Conformation of Purified ChrB

**Fig. 7:**
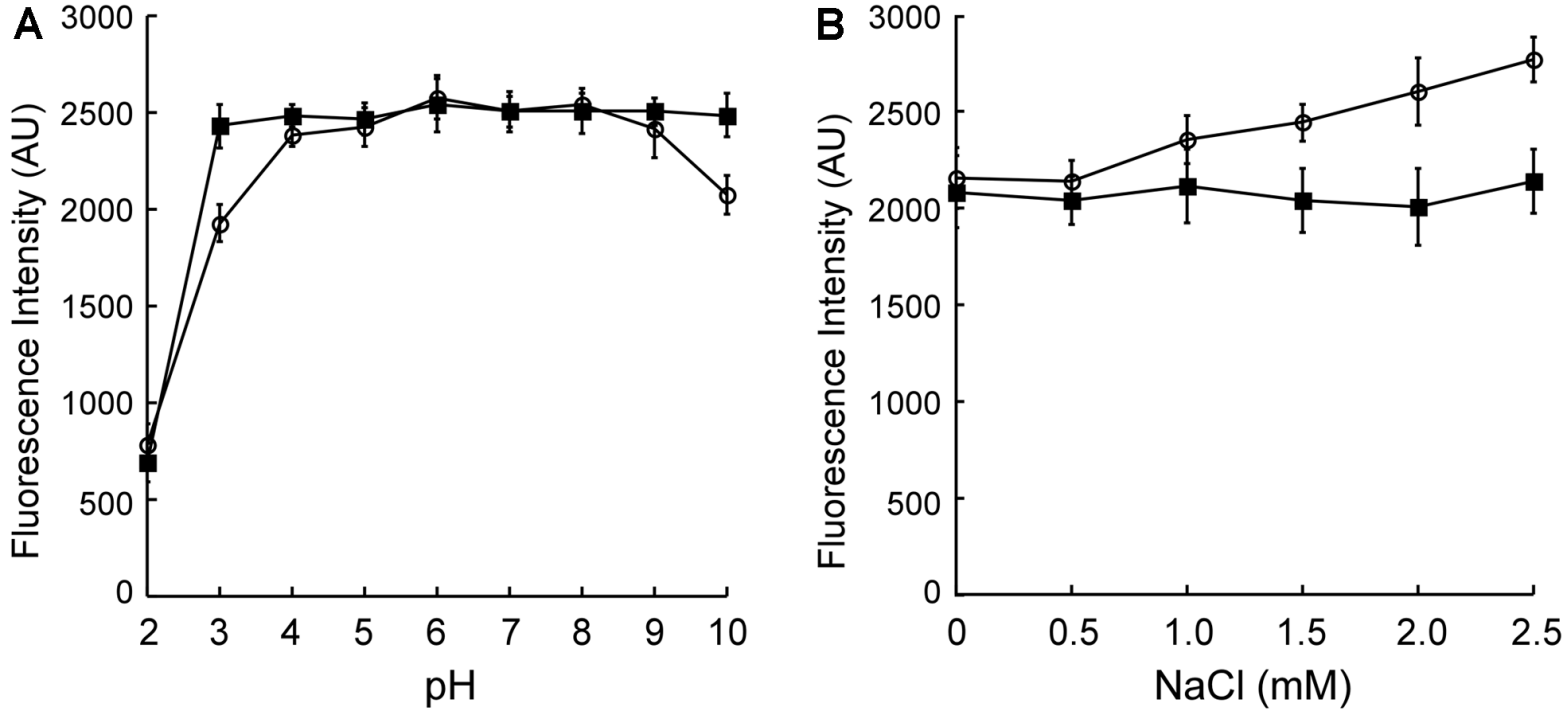
Effect of pH and salinity on the protein conformation of purified ChrB. (**A**) The purified ChrB protein (10 μM) in buffer was adjusted to different pH values before the intrinsic fluorescence of protein samples was measured (hollow circle). After all the samples were neutralized to pH ≈ 7, the intrinsic fluorescence of each sample was analyzed again (filled square). (**B**) The ChrB protein samples (10 μM in buffer containing 20 mM pH 7.0 Tris-HCl and 0, 0.5, 1.0, 1.5, 2.0, and 2.5 M NaCl, respectively) were also subjected to intrinsic fluorescence measurement (hollow circle). Then, all the protein samples were dialyzed into the buffer without NaCl (20 mM Tris-HCl, pH 7.0) before measuring the intrinsic fluorescence of each sample once more (filled square). The emission spectra were monitored from 300 to 500 nm with the excitation at 280 nm. Data are the means of three independent experiments, error bars represent the standard deviation.

To explain the effect of pH and salinity on the Cr(VI) adsorption capacity of *E. coli* expressing intracellular or surface-displayed ChrB, the intrinsic fluorescence emission of purified ChrB protein under different pH or salinity conditions was measured to monitor protein conformational changes. As shown in Fig. 7, the intrinsic fluorescence of ChrB protein was not significantly altered in the pH range of 4 to 9, and decreased by 23.1% or 17.3% at pH 3 or 10, respectively. However, at pH below 2, the protein was denatured, with the formation of visible white precipitates, and the intrinsic fluorescence intensity decreased dramatically. When the samples were subsequently neutralized, the intrinsic fluorescence of the acid- or alkali-treated protein samples recovered, except for the sample with pH 2, because the proteins in this sample had been irreversibly denatured. Similarly, we tested the intrinsic fluorescence of ChrB in the presence of increasing concentrations of NaCl, and we found that it gradually increased. After the removal of NaCl, the intrinsic fluorescence of all the NaCl-treated protein samples was restored. These results suggest that pH and salinity may influence the Cr(VI) adsorption capacity of *E. coli* expressing surface-displayed ChrB by modulating the protein conformation of ChrB.

## Discussion

Among the toxic heavy metals present in industrial wastewaters, chromium, especially Cr(VI), is considered extremely harmful. Therefore, effective removal of Cr(VI) is of vital importance. Because physicochemical methods are energy- and cost-intensive and result in the generation of secondary pollution, relevant biological treatment (bioremediation) techniques have garnered increasing attention.

Many microorganisms are capable of removing chromate ions [18]. There are two main types of bioremediation strategies utilized to treat Cr(VI)-contaminated wastewaters, biosorption and bioreduction. Biosorption is the removal of Cr(VI) ions from aqueous solutions by direct binding to bioadsorbents, whereas bioreduction involves the transformation of Cr(VI) to Cr(III), without altering the total amount of chromium. Biosorption has several advantages over bioreduction. First, biosorption is usually very fast and can be completed in a short time. For example, *Bacillus coagulans* was reported to adsorb 30.7–40.6 mg Cr(VI)/g dry weight within 1–2 h [19], and *Lantana camara* plants reportedly removed 98% of Cr(VI) from wastewater (initial concentration = 50 mg/l) in 30 min [20]. In this study, surface-displayed ChrB rapidly removed 99.1% of the total Cr(VI) in 2 h (Fig. S1). However, reduction of Cr(VI) to Cr(III) generally takes more than 24 h [21]. Further, reduced Cr(III) in wastewater can be re-oxidized to Cr(VI) under various conditions, for example, during chlorination of drinking water [22] or in chromium-contaminated sludge [23]. Therefore, Cr(VI) removal by biosorption is safer than bioreduction as it completely eliminates the risk of Cr(III) oxidization.

The efficiency of bacterial bioremediation can be increased in various ways. Genetic engineering can be utilized to enhance chromate adsorption or Cr(VI) reduction based on Cr(VI) resistance mechanisms. The Cr(VI) removal capabilities of the two *E. coli* strains engineered in this study were compared with respect to maximal adsorption capacity, adsorption efficiency, effect of pH, and salinity. The maximal adsorption capacity of pET- LOChrB/BL21 was about 235 μmol Cr(VI)/g dry weight, which is comparable to that of previously reported Cr(VI) bioadsorbents [24]. pET-LOChrB/BL21 cells removed nearly all Cr(VI) from wastewater (initial concentration = 0.5 mM) in 2 h, whereas the saturation point of adsorption with pET-ChrB-24h/BL21 occurred at 3–4 h, with only 42.9% of total Cr(VI) eliminated (Fig. S1). This is because intracellular adsorption requires transport of the metal ions across the cell membrane, and excess intracellular Cr(VI) would be exported out of the cell via resistance mechanisms [5]. Therefore, surface-displayed ChrB is substantially more effective for Cr(VI) adsorption than cytoplasmic expression under normal conditions.

However, effluents from the chromium electroplating industries are usually extremely acidic, with high levels of TDS. The data in Table S3 indicate that, under such conditions, Cr(VI) removal by pET-ChrB-24h/BL21 was greater than that by pET-LOChrB/BL21. It has been suggested that Cr(VI) in aqueous solution can form several species, such as Cr_2_O_7_^2−^, CrO_4_^2−^, and HCrO_4_^−^, which may have diverse protein-binding affinities. Speciation of Cr(VI) mainly depends on the pH of the solution [15]; thus, pH can influence Cr(VI) biosorption. More importantly, pH may influence metal binding by affecting the structural conformation of metal-binding proteins [16]. Our results also show that the pH and salinity can influence the protein conformation of ChrB (Fig. 7). However, *E. coli* cells can regulate cytoplasmic pH to protect biomolecules from extremely acidic or alkaline extracellular conditions [25]. The intracellular pH homeostasis of *E. coli* was attributed to pH-dependent catabolism and ion fluxes [26]. Furthermore, bacteria have evolved numerous systems to survive under high levels of salinity, which are responsible for metal homeostasis and resistance [27]. In this scenario, intracellular metal-binding proteins would be less susceptible to external variation in pH or ionic strength than surface-displayed proteins.

It should be noted that purified ChrB protein had a very weak binding affinity for Cr(VI) in vitro. The purified ChrB was treated with 10-fold excess of Cr(VI) solution and then repurified using a desalting column. Cr(VI) content analysis showed that each ChrB protein monomer could bind less than 1/10th molecule of Cr(VI). As purified ChrB protein reportedly can form oligomers (mainly dimers) [9], the putative chromate-binding residues (Arg180, Arg187, His229) were probably embedded upon oligomerization after the protein was purified from *E. coli* cells under aerobic condition. Thus, we attempted to reconstitute the purified ChrB with Cr(VI) in the presence of DTT, which reduced the protein to its monomeric form. However, DTT-treated ChrB still could not significantly bind Cr(VI), because the DTT also reduced Cr(VI) to Cr(III), which hardly has a binding affinity for ChrB (Fig. S3). Although oligomer formation can hinder purified ChrB from binding Cr(VI) in vitro, the majority of the proteins should be in the monomeric form in vivo, because reductases, e.g., enzymes of the thioredoxin antioxidant system [13] and glutathione reductase [28], are ubiquitously present in the cell. This was supported by the observation that approximately one molecule (1.13 ± 0.09) of Cr(VI) was detected in each His-tagged ChrB monomer when purified from *E. coli* cells that were pretreated with 5 mM Cr(VI), suggesting that ChrB is capable of binding Cr(VI) in vivo. Furthermore, fusion of ChrB to OmpA may avoid multimerization because the chimeric protein with a tightly folded conformation cannot be transported across the outer membrane [29], implying that ChrB displaying on the cell surface should be in the monomeric form. As the amount of intracellular ChrB produced after 6 h of induction was similar to that of surface-displayed protein after 24-h induction for the same cell number (Table S2), surface-displayed ChrB is estimated to bind approximately four atoms (4.25 ± 0.37) of Cr(VI) at maximum. While further investigations are required to elucidate the biochemical properties of ChrB and other metal-adsorbing proteins, our study provided useful strategies for the engineering of microorganisms to remove heavy metal pollutants.

## Supplemental Materials



Supplementary data for this paper are available on-line only at http://jmb.or.kr.
