# Supplementary material for: Removal of Chromium (VI) by Escherichia coli Cells Expressing Cytoplasmic or Surface-Displayed ChrB: a Comparative Study
Source: J Microbiol Biotechnol. 2020 Mar 20;30(7):996–1004. doi: 10.4014/jmb.1912.12030 (PMC9728187; doi:10.4014/jmb.1912.12030)
Supplement: Supplementary file 1 [file JMB-30-7-996-supple.pdf]

J. Microbiol. Biotechnol.
https://doi.org/10.4014/jmb.1912.12030

**Table S1** Primers used for construction of pET-ChrB, pET-ChrB-His and pET-LOChrB

| [illegible]er [illegible]e | Primer Sequence（5′→3′） |
|---|---|
| ChrB-1 | TTTAAGAAGGAGATATACCATGAACCTGCTGAGCCCGATCC |
| ChrB-2-his | TGGTGGTGGTGGTGGTGCTCCACGGTCAGGGTACCTTTTTCAAAGC |
| ChrB-2 | TGGTGGTGGTGGTGGTGCTCTCACACGGTCAGGGTACCTTTTTCAAAGC |
| Lpp-1 | TTTAAGAAGGAGATATACCATGAAAGCTACTAAACTGGTACTGGG |
| Lpp-2 | CGGGCCATTGTTGTTGATACCCTGATCGATTTTAGCGTTGCTGGAG |
| OmpA-1 | CGCTAAAATCGATCAGGGTATCAACAACAATGGCCCGACCCATG |
| OmpA-2 | GCTCAGCAGGTTCATGCCCGGGATACCACGGGTAGCGATTTCAGGAGTGATC |
| LOChrB-1 | ACCCGTGGTATCCCGGGCATGAACCTGCTGAGCCCGATCC |
| LOChrB-2 | TGGTGGTGGTGGTGGTGCTCTCACACGGTCAGGGTACCTTTTTCAAAGC |

**Table S2** Time-course analysis of protein expression of cytoplasmic and surface-displayed ChrB by quantitative western blotting

| Induction time (h) | pET-ChrB/BL21 | pET-LOChrB/BL21 |
|---|---|---|
| 2 | 52.65% | 27.83% |
| 6 | 109.82% | 48.11% |
| 12 | 242.71% | 75.34% |
| 24 | 321.54% | 100% |
| 36 | 330.25% | 96.26% |

*E. coli* cells (pET-ChrB/BL21 and pET-LOChrB/BL21) were grown in LB medium at 37 °C to an $OD_{600}$ of 0.6–0.7, and protein expression was induced by the addition of 200 μM IPTG. The cells were incubated at 25 °C for different incubation times (2 h, 6 h, 12 h, 24 h and 36 h, respectively). The cells were collected by centrifugation and resuspended in Tris buffers (final cell densities at $OD_{600} = 5$). For each sample, the induction of cytoplasmic or surface-displayed ChrB was identified by SDS-PAGE and the protein yield was compared by western blot using an anti-ChrB antibody. Band intensity on the western blot film was analyzed using ImageJ software. The relative protein yield (%) was defined as the ratio of the band intensity of each sample to that of pET-LOChrB/BL21 induced for 24 h (100%).

**Table S3** Cr(VI) and Cr(III) contents of industrial wastewaters before/after treated with ChrB expressed *E. coli* cells

| Sample | Intial concentrations | | pH | TDS (g/L) | After treated with pET-ChrB-24h/BL21 | | After treated with pET-LOChrB/BL21 | |
|---|---|---|---|---|---|---|---|---|
| | Cr (VI) (mg/L) | Cr (III) (mg/L) | | | Cr (VI) (mg/L) | Cr (III) (mg/L) | Cr (VI) (mg/L) | Cr (III) (mg/L) |
| 1 | 263 | 39.5 | 1.6 | 6.80 | 161 | 30.8 | 215 | 35.4 |
| 2 | 43.4 | 8.8 | 4.7 | 1.28 | 8.15 | 7.11 | 2.58 | 4.87 |
| 3 | 96.0 | 8.34 | 2.3 | 4.35 | 41.1 | 6.87 | 65.8 | 6.74 |
| 4 | 57.5 | 3.6 | 3.2 | 3.84 | 11.3 | 2.80 | 13.4 | 2.84 |
| 5 | 3.84 | 0.892 | 5.8 | 0.373 | 0.648 | 0.029 | 0.047 | 0.018 |
| 6 | 375 | 102 | 1.9 | 12.82 | 296 | 98 | 337 | 84 |
| 7 | 171 | 18.2 | 2.6 | 2.84 | 91.5 | 15.2 | 133 | 13.7 |
| 8 | 16.5 | 6.78 | 5.1 | 0.418 | 2.58 | 3.84 | 0.219 | 2.24 |
| 9 | 93.4 | 8.25 | 2.4 | 4.33 | 31.3 | 6.48 | 53.4 | 5.58 |
| 10 | 191 | 32.4 | 2.9 | 2.32 | 96.4 | 30.7 | 109 | 30.4 |
| 11 | 41.7 | 14.8 | 3.2 | 1.92 | 6.81 | 8.54 | 7.47 | 6.77 |
| 12 | 65 | 15.7 | 4.2 | 2.81 | 28.5 | 10.3 | 6.4 | 5.2 |

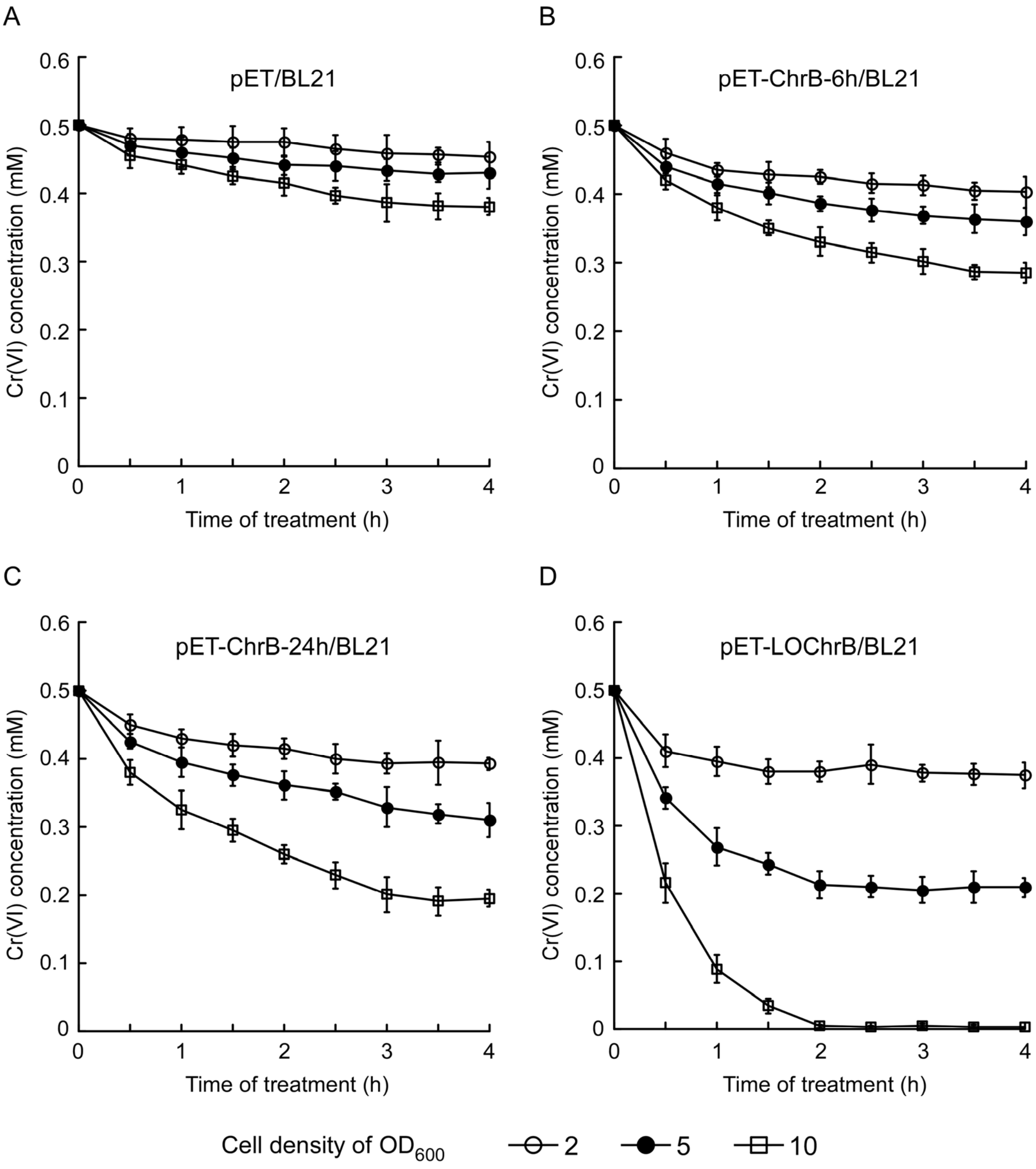


**Fig. S1** Removal of 1 mM $Cr^{6+}$ in aqueous solution by negative control cells (**a**, pET-BL21) and *E. coli* cells expressing intracellular ChrB (induced for 6 h in **b**, or 24 h in **c**) or surface-displayed ChrB (**d,** induced for 24 h). The cells were treated with $Cr^{6+}$ solutions at $OD_{600}$ = 2, 5, and 10. Supernatants of all samples were collected every 30 min for 4 h and analyzed for Cr(VI) content. Data are means of three independent experiments, error bars represent standard deviation.

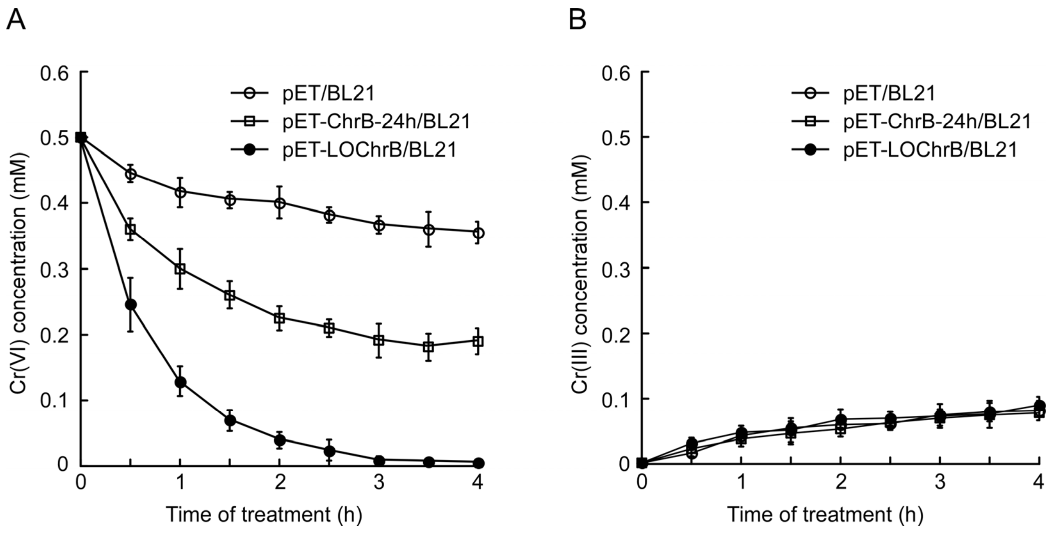


**Fig. S2** Determination of Cr(VI) and Cr(III) concentration of the water samples after treated by pET/BL21, pET-ChrB-24h/BL21 and pET-LOChrB/BL21. The cells ($OD_{600}$ = 10) were incubated with $Cr^{6+}$ solutions (initial concentration of 0.5 mM) for 4 h. Supernatants of all samples were collected every 30 min and analyzed for Cr(VI) and Cr(III) content. Data are means of three independent experiments, error bars represent standard deviation.

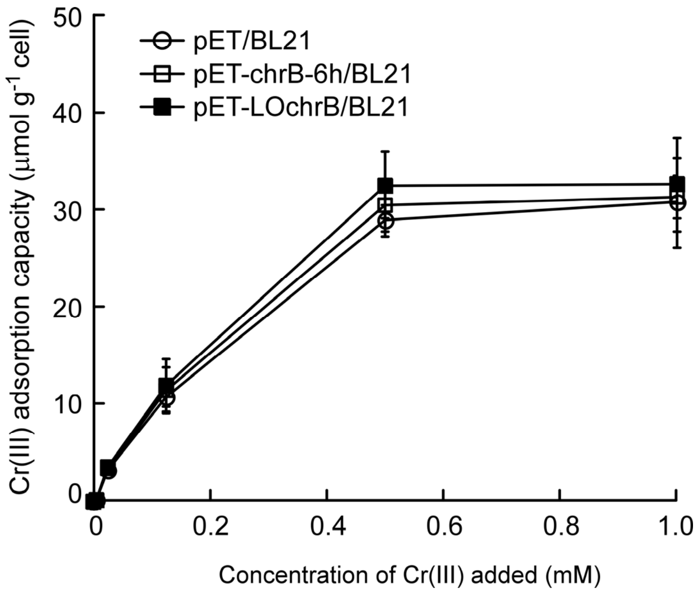


**Fig. S3** Cr(III) adsorption capacities of *E. coli* cells expressing surface-displayed ChrB (pET-LOChrB/BL21, induced for 24 h), intracellular ChrB (pET-ChrB-6h/BL21), and the negative control (pET/BL21). Cells were treated with increasing concentrations of $Cr^{3+}$ (0–1 mM). Data are the means of three independent experiments, error bars represent standard deviation.
